# Adropin and Endothelin-1 as Complementary Signals Associated with Early Vascular Aging in Middle-Aged Type 2 Diabetes

**DOI:** 10.3390/diseases14040140

**Published:** 2026-04-09

**Authors:** Rooban Sivakumar, Arul Senghor Kadalangudi Aravaanan, Vinodhini Vellore Mohanakrishnan, Janardhanan Kumar

**Affiliations:** 1 Department of Biochemistry, SRM Medical College Hospital and Research Centre, Faculty of Medicine and Health Sciences, SRM Institute of Science and Technology, SRM Nagar, Kattankulathur, Chengalpattu 603203, Tamil Nadu, India; 2 Department of General Medicine, SRM Medical College Hospital and Research Centre, Faculty of Medicine and Health Sciences, SRM Institute of Science and Technology, SRM Nagar, Kattankulathur, Chengalpattu 603203, Tamil Nadu, India

**Keywords:** early vascular aging, type 2 diabetes mellitus, adropin, endothelin-1, estimated pulse wave velocity

## Abstract

**Background**: Early vascular aging (EVA) is a common complication of type 2 diabetes mellitus. Early identification is crucial in middle-aged individuals with T2DM, as vascular stiffness can occur gradually for years before cardiovascular disease. However, EVA is rarely considered in routine care. Adropin is a vasoprotective peptide that may counter-regulate endothelin-1 (ET-1). Therefore, this study aims to examine the association between circulating adropin, ET-1, oxLDL, MMP-2, VEGFA, and EVA. **Methods**: This observational study included 300 adults aged 25–55 years (150 T2DM; 150 age/sex-matched controls). ePWV was calculated from age and mean blood pressure. EVA was classified using a residual-based, age-specific ePWV threshold derived from controls. Associations were tested using correlation and logistic regression. ROC and decision curve analyses were performed to evaluate diagnostic performance and clinical utility. **Results**: EVA prevalence was 38.6% overall, occurring in 7.3% of controls and increasing across T2DM with good and poor glycemic control (56.1% and 80.95%, respectively, *p* < 0.001). Compared with normal vascular aging, EVA showed lower adropin and higher ET-1, oxLDL and MMP-2, with lower VEGFA (all *p* < 0.05). In fully adjusted models, adropin (OR 0.991 per pg/mL; *p* < 0.001) and ET-1 (OR 1.017 per pg/mL, *p* = 0.005) remained independently associated with EVA. A combined adropin + ET-1 predictor improved discrimination (AUC 0.901, 95% CI 0.868–0.934), at a predicted-probability cutoff of 0.607, 78.7% sensitivity and 87.0% specificity. **Conclusions**: In middle-aged T2DM, EVA was associated with lower adropin and higher ET-1 in T2DM. These findings support an association between these biomarkers and the EVA phenotype.

## 1. Introduction

Type 2 diabetes mellitus (T2DM) accounts for 90% of all diabetes cases, and it is one of the most prevalent conditions globally. The incidence of diabetes is progressively escalating, with an estimated 425 million cases in 2017, projected to reach 783 million by 2045 [1,2]. In India, over 100 million individuals are estimated to have diabetes. This highlights the need for scalable methods to detect vascular complications early in midlife [3]. Approximately one third of patients with T2DM are at increased risk of both microvascular and macrovascular complications [4,5]. Importantly, T2DM also accelerates subclinical vascular injury via chronic hyperglycemia, insulin resistance, and related cardiometabolic stressors, leading to a progressive deterioration of arterial structure and function, which leads to premature vascular aging [6,7]. This problem is particularly urgent to address in middle-aged adults, where people with diabetes tend to develop cardiovascular disease (CVD) about 15–20 years earlier than those without diabetes [8].

Within this framework, early vascular aging (EVA) refers to premature structural and functional changes in arteries, resulting in aortic wall stiffening at a younger age than usual [9]. Arterial stiffness is a key indicator of vascular aging, which is measured through carotid-femoral pulse wave velocity (cfPWV) [10]. Although cfPWV serves as a reference standard, its direct measurement requires specialized devices and operator expertise, which restricts scalability. In this context, estimated PWV (ePWV) serves as a practical alternative for cohort studies, as it can be calculated from commonly available clinical variables. Recent studies also suggest that ePWV serves as a validated surrogate marker for assessing arterial stiffness and vascular aging [11,12]. Mechanistically, chronic hyperglycemia and insulin resistance can trigger oxidative stress and inflammation and impair endothelial nitric oxide (NO) production [13]. This will further increase advanced glycation end-product (AGE) production, which stiffens arteries through collagen cross-linking [14]. These interrelated pathways cause vasoconstriction, lipid-driven damage, and maladaptive remodeling in T2DM, suggesting EVA [15].

Numerous circulating indicators within this pathophysiologic framework reflect complementary aspects of diabetic vascular aging. Endothelin-1 (ET-1), a potent vasoconstrictor released by endothelial cells, is often elevated in diabetes and is significantly associated with the onset of vascular complications. Increased ET-1 often leads to endothelial dysfunction and dysregulation of vascular tone [16,17]. Simultaneously, diabetic dyslipidemia and oxidative stress lead to the production of oxidized low-density lipoprotein (oxLDL), which amplifies vascular inflammation and atherogenic signaling [18]. Uptake of oxLDL by macrophages leads to the formation of foam cells and triggers a cascade of pathways that result in the release of matrix metalloproteinases (MMPs), which are responsible for degrading the extracellular matrix in the vascular wall [19]. Meanwhile, dysregulated angiogenic signaling can disrupt endothelial repair, leading to abnormal production of vascular endothelial growth factor-A (VEGFA), representing impaired angiogenesis and vascular repair [20].

Emerging evidence suggests that adropin, a 76-amino-acid peptide coded by the energy homeostasis-associated (ENHO) gene, has become an important counter-regulatory peptide. It was first identified for its role in energy balance and lipid–glucose metabolism, and it is now being investigated for its vascular protective properties [21]. Experimental evidence suggests that adropin facilitates endothelial homeostasis, including signals associated with the activation of endothelial NO synthase, which aligns with a vascular protective phenotype [22]. Recent clinical studies also reveal that patients with T2DM and related metabolic disorders exhibit significantly lower circulating adropin levels compared to healthy individuals [23,24]. These observations suggest that adropin may serve as a biomarker and therapeutic target for vascular complications induced by diabetes.

As diabetic vascular aging is a multifaceted phenotype, single-marker methodologies may insufficiently encompass the biological diversity associated with EVA. Thus, a mechanistically integrated assessment that includes vasoactive stress, oxidative–atherogenic injury, structural remodeling and angiogenesis may provide a more comprehensive picture that causes early vascular aging in middle-aged T2DM. From this study, we aim to evaluate the association between adropin, ET-1, oxLDL, MMP-2, VEGFA and early vascular aging in T2DM patients, utilizing an age-referenced ePWV-based EVA phenotype. In addition to association analysis, we aim to perform exploratory analyses to assess how these markers differentiate between participants classified as EVA and normal vascular aging within the present dataset. We hypothesized that participants classified as having EVA would exhibit lower adropin and higher ET-1, oxLDL, and MMP-2 levels, together with altered VEGFA levels, compared with those without EVA. Here, we examine whether this integrated approach might enhance translational relevance for cardiovascular risk assessment and facilitate earlier diagnosis of EVA in midlife diabetes.

## 2. Materials and Methods

### 2.1. Study Design and Setting

This observational study was conducted at the SRM Medical College Hospital and Research Centre between July 2024 and October 2025. The study included a total of 300 participants, with 150 T2DM patients and 150 age- and sex-matched controls. Details of participant selection are given in Figure S1. Ethical approval was granted by the institutional Ethics Committee (Approval no. ECR/8856/INST/TN/2013/RR-19). All participants were thoroughly briefed about the research methods used in this study, and informed consent was obtained before their inclusion. The study strictly adhered to both the institution’s ethical standards and the Declaration of Helsinki standards. The sample size was determined based on the mean and standard deviation of adropin levels reported in previous research conducted by Wei W et al. [24], with an α of 0.05 and 90% power of the study.

### 2.2. Participant Selection

Participants were recruited from the Diabetology OPD and the Master Health Checkup using a purposive sampling approach. All participants were screened for diabetes, including medical history, anthropometry, blood pressure, and laboratory and clinical data. Individuals aged 25–55 years were enrolled in this study. Cases comprised individuals with known T2DM with a duration of ≥2 years. Based on the American Diabetes Association (ADA) and Research Society for the Study of Diabetes in India (RSSDI) recommendations, these participants were further stratified as having good glycemic control (HbA1c < 7.0%) or poor glycemic control (HbA1c ≥ 7.0%) for subgroup analysis [25,26]. Controls were healthy adults without diabetes, which was defined as HbA1c < 5.7% and fasting plasma glucose < 100 mg/dL [25], with no history of chronic illness or recent acute illness, not receiving any long-term medication, and with no history of cardiovascular disease. Participants with a history of cardiovascular disease, autoimmune disease, or chronic kidney disease, who were pregnant, or had a recent history of hospitalization, fever, or surgery within the preceding 45 days, were excluded from the study.

### 2.3. Blood Pressure Measurement and ePWV Estimation

An automatic digital sphygmomanometer (Omron HEM-7361T, Omron Healthcare Co., Ltd., Kyoto, Japan) with an atrial fibrillation (AFib) detector was used to assess the brachial blood pressure. All measurements were taken while seated after a 5 min rest period. The device was set up to an automatic three-reading protocol, capturing three continuous measurements at 30 s intervals. The average of these three readings was used as the brachial blood pressure value for each participant. Following this, mean blood pressure (MBP) was calculated as DBP + 0.4 (SBP-DBP). ePWV was determined from MBP and age using the following equations:

(1) ePWV = 9.58748315543126 − 0.402467539733184 * Age + 4.56020798207263 × 10^−3^*Age^2^ − 2.6207705511664 × 10^−5^*Age^2^*MBP + 3.1762450559276 × 10^−3^*Age*MBP − 1.83215068503821 × 10^−2^*MBP

(2) ePWV = 4.62 − 0.13*age + 0.0018*age^2^ + 0.0006*age*MBP + 0.0284*MBP.

Equation (1) is used for individuals with cardiovascular risk factors, and Equation (2) is used for individuals without cardiovascular risk factors. Non-smokers without metabolic syndrome components or a history of myocardial infarction or stroke were considered to have no cardiovascular risk factors [27,28].

### 2.4. Definition of Early Vascular Aging

EVA was defined using an age-referenced, residual-based approach adapted for the present cohort from the methodology described by Tomova GD et al. [29] and Tor V et al. [30]. In the control group, linear regression was used to develop an age-ePWV reference model, where age (years) was the predictor, and ePWV was the dependent variable:

*_Predicted_* ePWV = β0 + β1 × Age

This regression equation was used to compute the predicted ePWV for each participant. Unstandardized residuals were estimated as the difference between observed and predicted ePWV:

Residual = ePWV_observed_ − *_Predicted_* ePWV

The model fit was evaluated using R, R^2^, adjusted R^2^, ANOVA F-statistic, coefficient estimations with 95% confidence intervals, and standard error. Diagnostic residuals were examined using residual mean and standard deviation. Then, an upper-percentile threshold of the residual distribution in controls (80th percentile denoted as R80) was selected. Additionally, sensitivity analyses were performed using the 75th and 85th percentile residual thresholds. A participant-specific, age-adjusted ePWV cutoff was then derived by adding this residual threshold to the predicted ePWV.

ePWV _age-cutoff_ = *_Predicted_* ePWV + R80

Participants with observed ePWV at or above their age-specific cutoff were classified as having EVA (EVA = 1), and those below the cutoff were classified as having normal vascular aging (EVA = 0).

EVA = 1 if ePWV _observed_ ≥ ePWV _age-cutoff_, else EVA = 0

This age-specific thresholding procedure was applied uniformly to all participants in the dataset to generate the binary EVA phenotype for subsequent analyses. Additionally, 10-year cardiovascular risk was estimated for each participant using the QRISK3 score [31].

### 2.5. Sample Collection and Biochemical Analysis

Samples of 4 mL of peripheral venous blood were collected from all participants after an overnight fast of 8–10 h using appropriate vacutainer tubes. Samples were centrifuged and used for routine biochemical analyses. FPG, PPPG, and lipid parameters were assessed using a fully automated analyzer (Beckman Coulter DxC 700, Brea, CA, USA). HbA1c was measured via high-performance liquid chromatography (HPLC) utilizing Bio-Rad D-10 equipment. Total cholesterol and triglycerides were quantified via the CHOD-POD and enzymatic GPO–POD, respectively, whereas HDL-C and LDL-C were evaluated through a direct antibody inhibition. In addition, 2 mL of postprandial venous blood was collected for the estimation of postprandial plasma glucose (PPPG). The remaining serum was stored at −80 °C in a deep freezer for biomarker assays.

Serum adropin (Cat. OPK6444), endothelin-1 (ET-1) (Cat. OPK2444), oxidized low-density lipoprotein (oxLDL) (Cat. OPK5407), matrix metalloproteinase-2 (MMP-2) (Cat. OPK10203), and vascular endothelial growth factor-A (VEGFA) (Cat. OPK1129) were quantified using commercially available enzyme-linked immunosorbent assay (ELISA) kits (Origin Diagnostics & Research, Kerala, India) on a BioRad PR 4100 microplate reader (Bio-Rad Laboratories, Inc., Hercules, CA, USA). The manufacturer-reported analytical sensitivities and detection ranges were as follows: adropin 14.2 pg/mL (31.25–2000 pg/mL), ET-1 2.73 pg/mL (7.82–500 pg/mL), VEGFA 6.4 pg/mL (15.63–1000 pg/mL), oxLDL 0.51 ng/mL (1.57–100 ng/mL), and MMP-2 0.57 ng/mL (1.56–100 ng/mL). Every assay had manufacturer-reported intra-assay CVs < 8% and inter-assay CVs < 10%, according to the kit instructions. Concentrations were calculated using mean duplicate optical-density values for all samples. Following the manufacturer’s instructions, standard and blank wells were included in each test run. The ELISA procedure was carried out carefully in accordance with the guidelines provided by the manufacturers.

### 2.6. Statistical Analysis

Initial data collection and processing were carried out using Microsoft Excel. Subsequent statistical analyses were performed using SPSS 26 (Statistical Package for the Social Sciences, SPSS Inc., Chicago, IL, USA). The Kolmogorov–Smirnov test was used to determine the normality of the data. Continuous variables are presented as median (IQR: 25th–75th percentile) for non-normally distributed variables or mean ± SD for normally distributed variables, as appropriate, while categorical variables are presented as *n* (%). Between-group comparisons for two-group analyses were performed using the Mann–Whitney U test/independent *t*-test for continuous variables and the Chi-square test for categorical variables. For three-group comparisons, the Kruskal–Wallis test/one-way ANOVA was used as appropriate, with pairwise comparisons. Effect sizes for three-group comparisons were reported as epsilon squared (ε^2^) for Kruskal–Wallis tests. Spearman’s rank correlation was performed. Binary logistic regression was used to investigate the predictors associated with EVA. Circulating biomarkers and early vascular aging were examined using univariable and multivariable binary logistic regression. The following biomarkers were rescaled before modeling for interpretability: adropin to 100 pg/mL, ET-1 to 1 pg/mL, oxLDL to 10 ng/mL, MMP-2 to 10 ng/mL, and VEGFA to 10 units. Model diagnostics assessed multicollinearity using tolerance values and variance inflation factors. Linearity of continuous predictors in the logit was assessed using the Box–Tidwell approach. Receiver operating characteristic (ROC) analysis was used to evaluate diagnostic performance. Additionally, decision curve analysis (DCA) was conducted to evaluate clinical utility. *p* < 0.05 was considered statistically significant.

## 3. Results

### 3.1. Baseline Demographic and Clinical Characteristics

A total of 300 participants were analyzed. The median age was similar between the T2DM and control groups (48 vs. 49 years, *p* = 0.185). The T2DM group had a considerably higher BMI, suggesting many were overweight when compared to controls who remained within the normal BMI range (*p* < 0.001). Blood pressure was also elevated in the T2DM group, with median systolic/diastolic BP of 126/80 mmHg and 116/74 mmHg in controls (*p* < 0.001). Other baseline characteristics were almost similar between groups, including distribution of sex (*p* = 0.488), prevalence of smokers (*p* = 0.749) and alcohol use (*p* = 0.739). The median duration of diabetes in T2DM participants was around 4 years. Most of them were on metformin therapy (88%), with smaller subsets on other oral hypoglycemic agents (12%), insulin (2%), statins (10.6%), or antihypertensive drugs (15.3%) Table 1.

### 3.2. Impact of Glycemic Control on Cardiometabolic and Vascular Aging Indices

As expected, the diabetic profile varied significantly across controls and T2DM subgroups, with elevated fasting and postprandial glucose levels (*p* < 0.001). The HbA1c level of the poor glycemic control group is significantly higher when compared with those with good glycemic control and non-diabetic controls (*p* < 0.001). Lipid profiles and vascular metrics were also worsened with poorer glycemic control. Total cholesterol levels were elevated in individuals with T2DM compared to controls (*p* < 0.001), while no significant difference in total cholesterol was seen between well vs. poorly controlled T2DM patients (*p* = 0.579), whereas both triglycerides and LDL-C exhibited a glycemic-dependent increase. Atherogenic lipid ratios were significantly elevated in the poorly controlled T2DM group. Similarly, the TGL/HDL-C ratio was highest in poorly controlled T2DM when compared to well-controlled T2DM and controls (*p* = 0.002). ePWV also increased progressively from 7.21 m/s in controls to 7.72 m/s in well-controlled T2DM and 8.41 m/s in poorly controlled T2DM (*p* < 0.001). Likewise, the 10-year cardiovascular risk score (QRISK3) roughly doubled from controls to T2DM, reaching 18.6% in the poorly controlled subgroup (*p* < 0.001). These data indicate a markedly higher cardiovascular risk burden in T2DM, especially with poor glycemic control (Table 2 and Table S1). Effect sizes for three-group comparisons ranged from small to large, with the largest effects observed for glycemic variables, ePWV, adropin, ET-1, oxLDL, and MMP-2 (Table S2).

**Figure 1:**
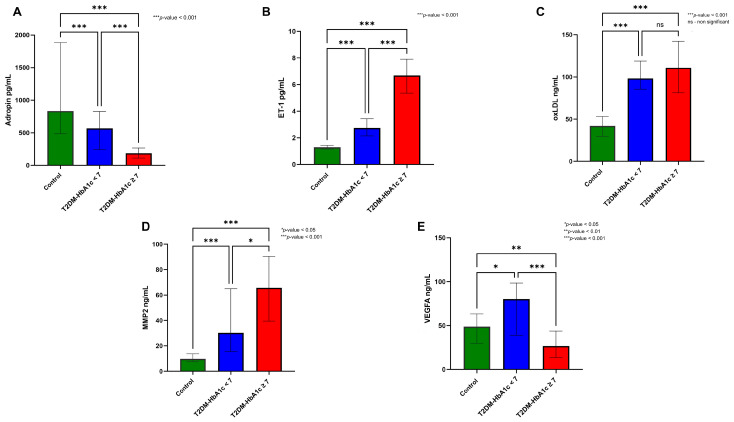
Group-wise comparison of circulating vascular aging markers across controls and T2DM subgroups stratified by glycemic control. Between-group differences were assessed using the Kruskal–Wallis test with pairwise comparison (**A**) adropin, (**B**) endothelin-1 (ET-1), (**C**) oxLDL, (**D**) MMP-2, and (**E**) VEGFA.

Figure 1 shows that adropin levels progressively decrease from controls (834.25 pg/mL) to well-controlled T2DM (566.79 pg/mL) and further to poorly controlled T2DM (185.34 pg/mL), with statistically significant differences among the groups (*p* < 0.001) (Figure 1A). In contrast, ET-1 levels increased with poor glycemic control, from 1.30 pg/mL in the control group to 2.74 pg/mL in well-controlled T2DM and 6.69 pg/mL in poorly controlled T2DM, showing significant differences among the groups (*p* < 0.001) (Figure 1B). oxLDL was significantly elevated in both T2DM subgroups (99.23 ng/mL and 110.81 ng/mL) compared to controls (41.87 ng/mL), while the changes in levels from well to poorly controlled T2DM were not significant (Figure 1C). Compared to controls, MMP-2 levels increased significantly in well and poorly controlled T2DM (9.80 ng/mL vs. 29.42 ng/mL vs. 65.67 ng/mL) (Figure 1D, *p* < 0.001). In well-controlled T2DM, VEGF levels were significantly higher (80.07 ng/mL) compared to controls (48.79, *p* < 0.05), whereas in poorly controlled T2DM, levels were lower (26.54 ng/mL) compared to both controls (*p* < 0.05) and well-controlled T2DM (Figure 1E).

### 3.3. Derivation of Age-Specific ePWV Threshold and EVA Classification

The age-expected ePWV for each participant was calculated using a linear regression model of age–ePWV. The fitted model was

*_Predicted_* ePWV = 3.029 + 0.089 × Age

The model showed strong fit (R = 0.884, R^2^ = 0.781, adjusted R^2^ = 0.780) and was statistically significant (F = 528.058, *p* < 0.001), with a standard error of estimate of 0.250. The intercept and age coefficient were both significant (intercept 95% CI 2.662–3.396; age coefficient 95% CI 0.082–0.097). Both the intercept and the age coefficient exhibited statistical significance (*p* < 0.001). Unstandardized residuals were determined by subtracting age-predicted ePWV from observed ePWV. The 75th, 80th, and 85th percentiles of the unstandardized control residual distribution were 0.193, 0.235, and 0.289 m/s, respectively. The 80th percentile of residuals (R80) was 0.2349868, and those with residuals greater than or equal to R80 were categorized as exhibiting early vascular aging (EVA). This residual-based definition is equivalent to using an age-specific ePWV threshold. Using different thresholds, sensitivity analyses indicated the predicted graded change in EVA prevalence, from 53.7% at R75 to 30.0% at R85, while maintaining group ordering (Table S3).

ePWV _age-cutoff_ = *_Predicted_* ePWV + R80 = 3.264 + 0.089 × Age

Participants with _observed_ ePWV values ≥ ePWV _age-cutoff_ were classified as EVA, and those below the cutoff were classified as normal. The mean age of our cohort is 47.7 years, which correlates to a cutoff of approximately 7.84 m/s. However, for each individual, the age-specific threshold was used to determine categorisation. Using this definition, 116 (38.6%) participants were classified as showing EVA. Incidence of EVA was low in healthy individuals (11/150, 7.3%) but was more common in T2DM, involving 56.1% of the well-controlled T2DM subgroup and 80.95% of the poorly controlled T2DM subgroup (*p* < 0.001) (Table S4). The prevalence of EVA was similar in men and women, with around 38.8% of men and 38.5% of women (*p* = 0.876; Table S5).

### 3.4. Biochemical and Vascular Profiles in Early Vascular Aging Phenotype

**Figure 2:**
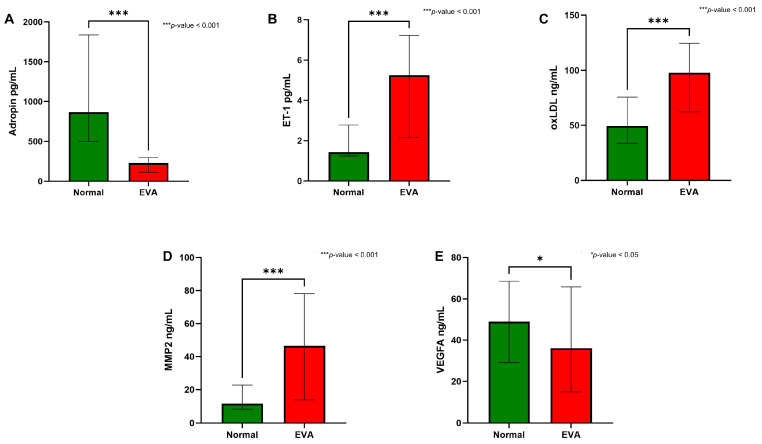
Comparison of circulating vascular aging markers between normal vascular aging and early vascular aging groups. Between-group differences were tested using Mann–Whitney U test. (**A**) Adropin, (**B**) endothelin-1 (ET-1), (**C**) oxLDL, (**D**) MMP-2, and (**E**) VEGFA.

Table 3 shows that the EVA group had poor glycemic control, with a median HbA1c of 7.4% and 5.4% in the normal vascular aging group (*p* < 0.001). Fasting and postprandial glucose were correspondingly higher in EVA (*p* < 0.001). Participants with EVA also demonstrated a more atherogenic lipid profile. Triglyceride levels were elevated in the EVA group compared to the normal vascular aging group (*p* < 0.001), and LDL-C was also slightly higher in the EVA group (*p* = 0.040). As expected, the EVA group had lower HDL-C than the normal vascular aging group (*p* = 0.012). The EVA group also exhibited a significantly higher 10-year cardiovascular risk (*p* < 0.001), indicating that participants classified as EVA had a more adverse overall cardiometabolic profile. Figure 2 further shows a clear biomarker gradient between vascular aging phenotypes. Adropin levels were markedly lower in the EVA group (227.89 pg/mL) than in the normal vascular aging group (867.21 pg/mL, *p* < 0.001) (Figure 2A), whereas ET-1 was significantly higher in EVA (5.25 pg/mL vs. 1.42 pg/mL, *p* < 0.001) (Figure 2B). Similarly, oxLDL and MMP-2 were both elevated in EVA (97.72 vs. 49.24 ng/mL and 46.54 vs. 11.71 ng/mL, respectively; both *p* < 0.001) (Figure 2C,D), indicating that the EVA phenotype was associated with an unfavorable circulating biomarker pattern. VEGFA showed a modest but significant decrease in EVA (36.11 vs. 48.92 ng/mL, *p* < 0.05) (Figure 2E), suggesting that this marker differed less strongly than the other biomarkers but still varied across vascular aging groups.

### 3.5. Adropin Correlates with Key Vascular Aging Indicators

**Figure 3:**
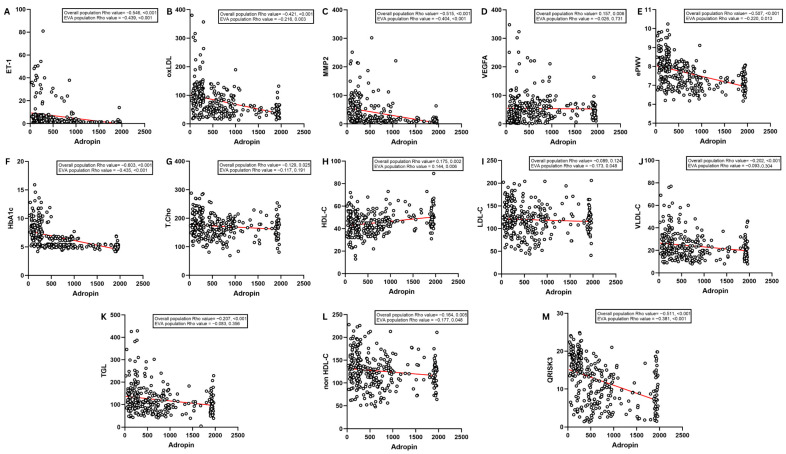
Correlations between circulating adropin and other parameters. Spearman rank correlation between adropin and (**A**) ET-1, (**B**) oxLDL, (**C**) MMP-2, (**D**) VEGFA, (**E**) ePWV, (**F**) HbA1c, (**G**) total cholesterol, (**H**) HDL-C, (**I**) LDL-C, (**J**) VLDL-C, (**K**) triglycerides, (**L**) non–HDL-C, and (**M**) QRISK3; *p* value < 0.05 is considered significant.

Figure 3 and Table S6 show that in the overall cohort, higher adropin correlated with lower ET-1 (ρ = −0.546), oxLDL (ρ = −0.421), MMP-2 (ρ = −0.515), and ePWV (ρ = −0.507) (*p* < 0.001). Adropin also showed strong inverse correlations with HbA1c (ρ = −0.603, *p* < 0.001) and was negatively correlated with atherogenic lipid indices such as VLDL-C (ρ = −0.202, *p* < 0.001), triglycerides (ρ = −0.207, *p* < 0.001), non-HDL-C (ρ = −0.164, *p* = 0.005), and total cholesterol (ρ = −0.129, *p* = 0.025). Meanwhile, the association with LDL-C was not significant (ρ = −0.089, *p* = 0.124). In contrast, adropin showed a weak positive correlation with VEGFA (ρ = 0.157, *p* = 0.006) and HDL-C (ρ = 0.175, *p* = 0.002). Importantly, within the EVA subgroup, adropin is inversely correlated with ET-1 (ρ = −0.439, *p* < 0.001), oxLDL (ρ = −0.216, *p* = 0.003), MMP-2 (ρ = −0.404, *p* < 0.001), ePWV (ρ = −0.220, *p* = 0.013), HbA1c (ρ = −0.435, *p* < 0.001), and QRISK3 (ρ = −0.381, *p* < 0.001). In the EVA subgroup, adropin remained positively associated with HDL-C (ρ = 0.144, *p* = 0.006) and showed small inverse associations with LDL-C (ρ = −0.173, *p* = 0.048) and non-HDL-C (ρ = −0.177, *p* = 0.048), whereas associations with VEGFA, total cholesterol, VLDL-C, and triglycerides were not significant (Figure 3A–M). A detailed correlation matrix for all study variables is presented as a heatmap in Figure S2.

### 3.6. Univariable and Multivariable Logistic Regression of EVA Predictors

Table 4 shows that an increase in age (OR = 1.207, 95% CI 1.142–1.275, *p* < 0.001) and BMI (OR = 1.156, 95% CI 1.052–1.270; *p* = 0.003) was associated with higher odds of EVA. HbA1c also showed approximately two-fold higher odds of EVA per unit increase (OR = 2.149, 95% CI 1.758–2.627; *p* < 0.001). Among circulating biomarkers, adropin was inversely associated with EVA, indicating lower adropin levels in participants with EVA (B = −1.981, *p* < 0.001). In contrast, higher levels of ET-1 (OR = 1.084, 95% CI 1.042–1.128; *p* < 0.001), oxLDL (OR = 1.024, 95% CI 1.017–1.031, *p* < 0.001), and MMP-2 (OR = 1.021, 95% CI 1.013–1.028, *p* < 0.001) were each significantly associated with increased odds of EVA. Sex and VEGFA were not significantly associated with EVA in univariable models. Following this, multivariable logistic regression demonstrated that adropin and ET-1 were the only biomarkers independently associated with EVA across all adjusted models (Table 5). In the crude model (Model 1), each 100 pg/mL increase in adropin was associated with 0.606-fold lower odds of EVA (OR = 0.606, 95% CI 0.548–0.740, *p* < 0.001). In contrast, each 1 pg/mL increase in ET-1 was associated with 1.014-fold higher odds of EVA (OR = 1.014, 95% CI 1.005–1.024, *p* = 0.003). These relationships remained statistically significant after adjustment for age, sex, and HbA1c (Model 2). In the fully adjusted model, both adropin and ET-1 remained independently associated with EVA. Each 100 pg/mL increase in adropin was associated with 0.405-fold lower odds of EVA (OR = 0.405, 95% CI 0.331–0.670, *p* < 0.001), while each 1 pg/mL increase in ET-1 was associated with 1.017-fold higher odds of EVA (OR = 1.017, 95% CI 1.005–1.029, *p* = 0.005). Also, the final adjusted logistic regression model was significant overall (Omnibus χ^2^ = 181.917, df = 5, *p* < 0.001). Model calibration was acceptable on the Hosmer–Lemeshow test (χ^2^ = 12.399, df = 8, *p* = 0.134). The model showed Cox and Snell R^2^ = 0.455 and Nagelkerke R^2^ = 0.611. OxLDL, MMP-2, and VEGFA were not independently associated with EVA (Table 5). Multicollinearity was also low, with tolerance values ranging from 0.707 to 0.928 and VIFs ranging from 1.078 to 1.414, indicating no evidence of problematic collinearity among predictors (Table S7). Additionally, Box–Tidwell testing showed no significant interaction terms for the continuous predictors (all *p* > 0.05), indicating no evidence of violation of the linearity-in-the-logit assumption (Table S8).

### 3.7. Diagnostic Accuracy of Biomarkers for EVA

**Figure 4:**
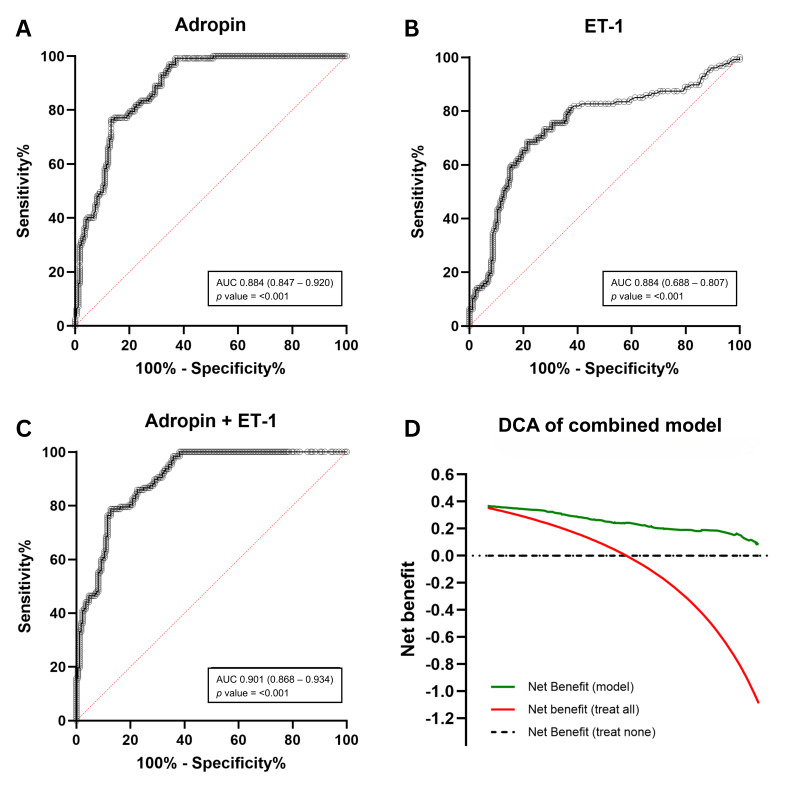
Diagnostic performance of adropin and endothelin-1 (ET-1) for early vascular aging. ROC shows the ability of (**A**) adropin, (**B**) ET-1, and (**C**) the combined adropin + ET-1 logistic predictor to differentiate EVA from normal vascular aging. (**D**) Decision curve analysis (DCA) for the combined model shows net benefit across threshold probabilities compared with “treat-all” and “treat-none” strategies.

As shown in Figure 4, ROC analyses revealed that at an optimal cutoff of 677 pg/mL (Youden’s J = 0.631), adropin achieved 76.4% sensitivity and 86.7% specificity, with PPV 78.4%, NPV 82.7%, LR+ 5.74, and an overall accuracy of 81.6%. Likewise, ET-1 at a cutoff of 3.13 pg/mL (Youden’s J = 0.471) yielded 68.5% sensitivity and 78.6% specificity, with PPV 66.9%, NPV 79.8%, LR+ 3.20, and 74.7% accuracy. A combined predictor derived from binary logistic regression (adropin + ET-1) provided the best outcome model (AUC 0.901, 95% CI 0.868–0.934; *p* < 0.001). Using a predicted-probability cutoff of 0.607 (Youden’s J = 0.660), the combined model achieved 78.7% sensitivity and 87.0% specificity, with PPV 79.6%, NPV 86.0%, LR+ 6.2, and 84.0% accuracy (Table S9; Figure 4A–C). Consistent with these findings, decision curve analysis (DCA) demonstrated that the combined model provides a greater net benefit than the “treat-all” and “treat-none” strategies, thereby supporting its potential clinical utility for risk stratification (Figure 4D). However, because the same dataset was used for model derivation and evaluation, these findings may overestimate performance and require external validation.

Both adropin and ET-1 also predicted glycemic control in the diabetic subgroup analysis (Table S10, Figure S3). Adropin showed an AUC of 0.815 (95% CI 0.744–0.887, *p* < 0.001) at a cutoff of 502 pg/mL (Youden’s J = 0.574) with 95.2% sensitivity and 62.1% specificity (PPV 76.4%, NPV 90.9%, LR+ 2.51), with 80.7% accuracy. ET-1 yielded an AUC of 0.823 (95% CI 0.741–0.904, *p* < 0.001), and at a cutoff of 3.44 pg/mL (Youden’s J = 0.761) demonstrated 98.8% sensitivity and 77.3% specificity (PPV 84.9%, NPV 98.0%, LR+ 4.35), with 89.4% accuracy.

## 4. Discussion

### 4.1. Key Findings and Related Studies

Early vascular aging in T2DM is increasingly linked to cardiovascular risk. Diabetes can essentially cause the artery to age six to fifteen years faster [32]. To support earlier risk stratification, we evaluated the association between circulating adropin, endothelin-1, oxLDL, MMP-2, and VEGFA and EVA in middle-aged T2DM patients. To our knowledge, this is the first study to combine an age-referenced ePWV EVA definition with an adropin-axis multi-marker panel in a middle-aged population to reveal accelerated vascular aging in T2DM. As mentioned earlier, our cohort had a mean age of 47.7 with an ePWV cutoff of 7.84 m/s. This aligns with a recent large cohort study on middle-aged individuals, which reported 7.407 m/s as a cutoff associated with incident metabolic syndrome (sensitivity 74.3%, specificity 46.4%) [33]. Similar biomarker patterns have been described in other vascular-risk states, although those cohorts are not directly comparable to our middle-aged T2DM population [34]. In addition to stiffness-based EVA phenotyping, we evaluated the estimated 10-year CVD risk using QRISK3, a multivariable risk algorithm established in primary care and recommended for formal risk assessment in individuals aged 25–84, including individuals with type 2 diabetes [31]. In our cohort, the EVA group exhibited a substantially higher QRISK3 score, indicating that ePWV-defined EVA co-segregates with a clinically meaningful elevation in estimated global CVD risk. This is consistent with a recent study reporting that higher QRISK3 scores are associated with subclinical vascular aging indicators, such as PWV, cfPWV, and carotid measurements [35].

Further, our findings demonstrate that adropin levels were significantly decreased in the EVA group, while ET-1, oxLDL, and MMP-2 were elevated. These results align with a study by Jurrissen et al., who reported that obesity and T2DM are associated with reduced circulating adropin and increased arterial stiffness in both humans and mice [36]. They further demonstrated a causal relationship by showing that adropin deficiencies result in increased arterial stiffness in animal models, hence strengthening the association between “hypoadropinemia” and arterial stiffness [36]. Consistent with this, a recent clinical study also indicated that diabetic individuals with macrovascular problems exhibited considerably lower serum adropin levels compared to healthy controls [37]. This is consistent with an association between lower adropin levels and adverse vascular phenotypes reported in T2DM.

Most studies, including ours, report lower adropin levels in patients with diabetes and especially in those with vascular complications. However, some reports have noted no significant correlation between adropin and metabolic parameters [38]. A study by Palizban et al. found higher adropin levels in T2DM patients when compared to controls with certain polymorphisms. In that study, the T2DM cohort had a longer disease duration when compared to ours. The authors further hypothesized that chronic disease could trigger an adaptive upregulation of adropin in some individuals or that genetic factors could modify its baseline levels [38]. Such contradictions suggest that population differences, disease stage, and methodological factors can affect adropin findings. A recent study in cardiac surgery patients found that higher serum big ET-1, a precursor of ET-1, was strongly associated with increased carotid-femoral pulse wave velocity and arterial stiffness [39]. Elevated ET-1 levels were also seen in individuals with diabetic CAD [40] and were also observed in individuals with increased arterial stiffness [41], indicating endothelial injury and adverse vascular remodeling. This finding aligns with our observation of higher ET-1 in EVA, underlining that even in asymptomatic middle-aged diabetics, elevated ET-1 was associated with the EVA phenotype in our cohort.

Beyond adropin and ET-1, our findings support the association of oxidative stress and matrix remodeling with arterial stiffness. We found oxLDL to be higher in EVA subjects, mirroring the well-established role of oxidized lipoproteins in atherosclerosis and vascular dysfunction [42]. In T2DM, LDL-C is more susceptible to oxidative modification, and plasma oxLDL levels are therefore often higher [43]. This pro-oxidative environment has been linked to endothelial damage and arterial stiffness in the prior literature. A recent cross-sectional study in T2DM revealed that an oxidative damage marker independently predicted increased vascular stiffness, linking systemic oxidative stress with arterial aging [44,45]. Similarly, we noted elevated MMP-2 in the EVA group. A study conducted by Stabouli S et al. revealed that MMP-2 is directly associated with arterial stiffness, which aligns with our findings [46]. Additionally, in vitro and animal studies suggest that the upregulation of MMP-2 can lead to increased arterial stiffening by degrading elastin and collagen, as well as promoting vascular calcification through bone morphogenetic protein-2 (BMP-2) signaling [47]. Finally, VEGF-A levels differed significantly between EVA and non-EVA participants with diabetes, which may be consistent with altered angiogenic signaling. A recent study by Sun et al. reported that VEGF levels increase early in dysglycemia in relation to insulin resistance [48]. Our inverted U-shaped pattern suggests an initial compensatory angiogenic response in well-controlled T2DM, which reduces with progression to EVA or poor glycemic control. Again, Chen et al. found that DFU had lower VEGFA than diabetes controls and that increased VEGFA reduced DFU risk [49]. This is consistent with the prior literature linking lower VEGF-A to vascular complications in advanced disease, although the present study did not assess underlying mechanisms directly.

### 4.2. Potential Biological Links Between Adropin, ET-1, and EVA

Although this cross-sectional study cannot demonstrate mechanisms, directionality, or causation, the previous experimental and clinical literature provides a biological framework that may help explain the observed relationships between adropin, ET-1, and early vascular aging. Prior studies suggest that adropin enhances endothelial nitric oxide bioavailability by stimulating eNOS phosphorylation at Ser^1177^ via activation of VEGF receptor 2 (VEGFR2) and downstream PI3K/Akt and ERK1/2 signaling pathways [22]. This leads to increased nitric oxide (NO) bioavailability, which in turn improves endothelium-dependent vasodilation and vascular health [50]. Adropin also enhances pro-angiogenic and repair processes in the vasculature by increasing endothelial cell proliferation, migration, and capillary network formation in vitro [50]. In contrast, low adropin levels have been associated with endothelial dysfunction. An in vitro study by Yang et al. revealed that adropin reduces endothelial permeability and inflammatory signaling via the NO pathway [51]. Similarly, in a middle-aged vascular dysfunction study, adropin was measured together with ET-1, NO, and flow-mediated dilation (FMD). This study revealed that patients showed lower adropin levels, higher ET-1 levels, and lower FMD, with adropin levels being positively associated with FMD [52]. These direct effects suggest that adropin acts as a vasoprotective factor. However, these studies differ substantially from the present dataset in phenotype definition and measurement approach.

ET-1 is mainly produced by endothelial cells and is one of the most potent vasoconstrictors. It exerts its biological effects via ETA and ETB receptors on vascular smooth muscle and endothelium [53]. ET-1 activates ETA receptors, which induce vasoconstriction and promote the proliferation, fibrosis, and inflammation of VSMCs, whereas ETB receptors remove ET-1 and mediate some vasodilatory feedback via NO release. In the context of vascular aging, it increases hemodynamic load, generates vascular oxidative stress, triggers pro-inflammatory signaling, stimulates VSMC migration and proliferation, and promotes extracellular matrix deposition and fibrosis [53]. Indeed, chronic low-grade ET-1 elevation has been reported in elderly individuals, and endothelin-mediated vasoconstrictor tone increases with age in healthy adults [54]. Emerging data suggest an inverse relationship between adropin and ET-1, which form a counter-regulatory axis. Similar patterns have been described in other vascular-risk states, such as hypertension, where lower plasma adropin and higher ET-1 were observed compared to normotensives, and, importantly, plasma adropin and ET-1 levels were inversely correlated. Further, multivariate analysis in that study identified low adropin and high ET-1 as independent predictors of high blood pressure [55]. This suggests that reduced adropin and increased ET-1 together drive vascular dysfunction. In terms of vascular regulation, both adropin and ET-1 directly antagonize each other’s effects. Adropin’s potential to enhance NO production can reduce ET-1-mediated vasoconstriction. NO is recognized for its ability to inhibit the transcription and secretion of endothelin-1 in endothelial cells, functioning as part of a feedback mechanism that regulates vascular tone [56]. Thus, with sufficient adropin levels, eNOS-derived NO can control ET-1 activity, preserving vasodilation. On the other hand, excess ET-1 can diminish the adropin/NO pathway. Chronic ET-1 exposure reduces eNOS expression and NO availability [16] and also promotes oxidative stress that scavenges NO. Any disruption to this two-way antagonism of the adropin–ET-1 axis can be a plausible driver of EVA; however, this cannot be determined from the present cross-sectional design. Further experimental studies are needed.

### 4.3. Translational Implications and Future Research Directions

Our findings have several translational implications, notably for adropin and ET-1 as early T2DM vascular-risk factors. Low circulating adropin may indicate vascular aging or endothelial health decline among diabetic patients if validated in larger cohorts. Clinical adropin measurement is not currently accessible, although research assays could eventually be standardized. A T2DM patient with an unusually low adropin level for their age may be at risk for EVA and its complications. These patients may benefit from more active risk factor therapy, such as maintaining better glycemic control, blood pressure reduction, and lifestyle changes to decrease the progression of arterial stiffness. A clear next step is to conduct longitudinal studies to determine the predictive value of these biomarkers for EVA and cardiovascular outcomes. Such longitudinal validation is essential before proposing any biomarker in routine risk stratification. There is also a need for mechanistic interventional studies, testing whether modifying these biomarker pathways changes the course of vascular aging. Further research is also needed to explore the precise molecular interplay among these biomarkers. Finally, studies should include diverse populations and earlier stages of disease. EVA can begin even in pre-diabetes or youth with obesity. Investigating these biomarkers in younger T2DM patients, type 1 diabetes, or metabolic syndrome without diabetes might reveal how early these alterations occur. The potential impact of preexisting pharmacotherapy on circulating biomarker levels is an additional factor to consider. Previous studies suggest that metformin may enhance endothelial function and, in certain clinical contexts, decrease ET-1-related endothelial activation [57,58]. Following this statin therapy has also been linked to reduced circulating ET-1 levels, whereas antihypertensive treatments with agents like valsartan or amlodipine have been noted to elevate circulating adropin concentrations [59]. Furthermore, GLP-1 receptor agonist therapy may be crucial in this setting, since small interventional research including obese males with T2DM indicated a significant elevation in plasma adropin following 3 months of liraglutide administration. Despite the inclusion of medication usage in the fully adjusted model, residual direct effects of medication on circulating ET-1 and adropin cannot be ruled out and may have somewhat influenced the biomarker profile found in the diabetic cohort [60].

Our study has several strengths. A well-characterized middle-aged cohort comprising controls and T2DM individuals stratified by glycemic control allowed vascular aging evaluation across clinically relevant categories. A supplementary biomarker panel, age-referenced EVA definition, and standardized analytical methodologies were explored. Finally, multivariable and sensitivity analyses supported association-based conclusions.

There are some limitations to be addressed in this study. First, this study is cross-sectional in nature, which limits our ability to infer causality. Participants were recruited using a purposive, hospital-based sampling strategy from a single center, which introduces the possibility of selection bias. Second, our study population was predominantly from a single ethnic background and geographic region, which limits generalizability. External validation in population-based and multi-center cohorts is needed before broader generalization. Finally, the measurement of arterial stiffness by ePWV is practical for large samples, but it is an indirect surrogate for arterial stiffness. Future studies can be conducted via direct measurements (cfPWV) by tonometry, which is considered the gold standard. Also, ePWV incorporates mean blood pressure in its calculation; part of the observed ET-1 association may reflect blood-pressure-related variation within the surrogate EVA definition. However, we believe that ePWV was sufficient to stratify early vascular aging in our sample because it has been validated and correlates with outcomes [11].

## 5. Conclusions

Middle-aged T2DM patients with early vascular aging exhibited elevated ET-1, oxLDL, and MMP-2 and decreased adropin, along with alterations in VEGFA, which are consistent with oxidative damage, matrix remodeling, and endothelial dysfunction. These findings suggest that adropin and ET-1 can be used as early screening markers. Also, these findings should be interpreted as associative and do not establish causality.

## Figures and Tables

**Table 1:** Baseline demographic and clinical characteristics of study participants.

Variable	Control (*n* = 150)	T2DM (*n* = 150)	*p* Value
Age, years	48 (43, 52)	49 (45, 53)	0.185
25–35 years, *n* (%)	4 (2.7)	5 (3.3)	0.54
36–45 years, *n* (%)	52 (34.7)	41 (27.3)	
46–55 years, *n* (%)	94 (62.6)	104 (69.4)	
Male, *n* (%)	79 (52.6)	73 (48.6)	0.488
BMI, kg/m^2^	22.21 (21.5, 22.8)	24.38 (22.3, 26.2)	**<0.001**
Normal, *n* (%)	143 (95.3)	89 (59.3)	**<0.001**
Overweight, *n* (%)	7 (4.7)	52 (34.7)	
Obese, *n* (%)	0	9 (6)	
SBP, mmHg	116 (112, 120)	126 (120, 132)	**<0.001**
DBP, mmHg	74 (71, 76)	80 (76, 88)	**<0.001**
MBP, mmHg	88 (85, 90)	95 (91, 104)	**<0.001**
Smoking (Yes), *n* (%)	24 (16)	22 (14.6)	0.749
Alcohol (Yes), *n* (%)	22 (14.6)	20 (13.3)	0.739
Diabetes duration, years	-	4 (3, 6)	-
Metformin use, *n* (%)	-	132 (88)	-
Other OHA use, *n* (%)	-	18 (12)	-
Insulin use, *n* (%)	-	3 (2)	-
Statin use, *n* (%)	-	16 (10.6)	-
AHD use, *n* (%)	-	23 (15.3)	-

Mann–Whitney U test has been performed for continuous variables and Chi-square test for categorical variables. Data presented as medians (IQR = 25th quartile, 75th quartile); *p* value < 0.05 is considered significant; BMI—body mass index, SBP—systolic blood pressure, DBP—diastolic blood pressure, MBP—mean blood pressure, OHA—oral hypoglycemic agents, AHD—antihypertensive drugs.

**Table 2:** Comparison of biochemical and vascular aging indices among controls and T2DM subgroups stratified by glycemic control.

Parameter	Control (n = 150)	T2DM–HbA1c < 7 (n = 66)	T2DM–HbA1c ≥ 7 (n = 84)	*p^a^*-Value	*p^b^*-Value
***Diabetic Profile***					
FPG, mg/dL	91 (86, 95)	112.5 (103.5, 129)	158 (134, 205.5)	**<0.001**	**<0.001**
PPPG, mg/dL	116 (110, 123.25)	148 (129.75, 186)	242 (212, 277)	**<0.001**	**<0.001**
HbA1c, %	5.2 (5, 5.4)	6.5 (6.3, 6.8)	8.85 (7.75, 10.4)	**<0.001**	**<0.001**
eAG	103 (97, 108)	140 (134, 148)	207.5 (175.5, 252)	**<0.001**	**<0.001**
**Lipid Profile**					
Total cholesterol, mg/dL	154 (130, 181.5)	179 (163, 200.5)	182 (158.75, 220)	**<0.001**	0.579
Triglycerides, mg/dL	97 (71, 123.75)	109.5 (86, 141.5)	138.5 (108.25, 192)	**<0.001**	**0.021**
HDL-C, mg/dL	46 (41, 52)	44.5 (40, 48)	43 (36, 50)	0.61	0.511
LDL-C, mg/dL	112.5 (91, 128.5)	118.5 (102, 137)	128 (109.25, 158.75)	**<0.001**	**0.047**
VLDL-C, mg/dL	19 (14, 25)	22 (17, 28)	27 (21.75, 35.75)	**<0.001**	**0.001**
Non-HDL-C, mg/dL	115 (93.25, 133)	130 (105.75, 149)	142 (117, 173)	**<0.001**	**0.006**
TC/HDL-C	3.56 ± 0.82	3.89 ± 0.90	4.42 ± 0.98	**<0.001**	**0.001**
LDL-C/HDL-C	2.49 ± 0.73	2.74 ± 0.74	3.09 ± 0.82	**<0.001**	**0.022**
TGL/HDL-C	2.4 ± 1.03	2.79 ± 1.17	3.69 ± 1.25	**<0.001**	**0.002**
**Risk Metrics**					
ePWV, m/s	7.21 (6.86, 7.7)	7.72 (7.26, 8.07)	8.41 (7.69, 8.86)	**<0.001**	**<0.001**
QRISK3, %	7.05 (4.1, 8.5)	14.4 (12.3, 16.5)	18.6 (16.17, 19.9)	**<0.001**	**<0.001**

*p^a^* (Kruskal–Wallis test and ANOVA) represents the difference between 3 groups, *p^b^* (Mann–Whitney U test and independent *t*-test) represents the difference between good glycemic control and poor glycemic control; data presented as median (IQR = 25th quartile, 75th quartile) and mean ± SD; *p* value < 0.05 is considered significant; FPG, fasting plasma glucose; PPPG, postprandial plasma glucose; eAG, estimated average glucose; HDL-C, High-Density Lipoprotein Cholesterol; LDL-C, Low-Density Lipoprotein Cholesterol; VLDL-C, Very-Low-Density Lipoprotein Cholesterol; ePWV, estimated pulse wave velocity.

**Table 3:** Comparison of biochemical and cardiovascular risk metrics between normal vascular aging and early vascular aging groups.

Variable	Normal VA (n = 184)	Early VA (n = 116)	*p* Value
***Diabetic Profile***			
FPG, mg/dL	95 (88, 103.5)	126 (100, 166)	**<0.001**
PPPG, mg/dL	121 (113, 139.5)	198 (127, 249)	**<0.001**
HbA1c, %	5.4 (5.1, 6.2)	7.4 (6.5, 9.4)	**<0.001**
eAG	108 (100, 131)	166 (129, 223)	**<0.001**
**Lipid Profile and Risk Metrics**			
Total cholesterol, mg/dL	166 (138.5, 185.5)	178 (153, 216)	**<0.001**
Triglycerides, mg/dL	100 (76, 133)	122 (91, 165)	**<0.001**
HDL-C, mg/dL	46 (41, 52)	43 (37, 49)	**0.012**
LDL-C, mg/dL	117 (95, 132)	120 (105, 150)	**0.040**
VLDL-C, mg/dL	20 (15, 27)	24 (18, 33)	**<0.001**
Non-HDL-C, mg/dL	121 (96.25, 139)	131 (111, 160.5)	**<0.001**
TC/HDL-C	3.67 ± 0.88	4.1 ± 0.99	**<0.001**
LDL-C/HDL-C	2.57 ± 0.78	2.90 ± 0.79	**<0.001**
TGL/HDL-C	2.50 ± 1.30	3.34 ± 1.25	**<0.001**
QRISK3, %	9.7 (5.5, 13.75)	17.5 (14.4, 19.5)	**<0.001**

Mann–Whitney U test and independent *t*-test were performed, Data presented as median (IQR = 25th quartile, 75th quartile) and mean ± SD; *p* value < 0.05 is considered significant; VA—vascular aging.

**Table 4:** Univariable logistic regression analysis of predictors associated with early vascular aging.

Predictor	B	S.E.	*p* Value	Exp(B)-OR	95% CI
Age	0.188	0.028	**<0.001**	1.207	1.142–1.275
BMI	0.145	0.048	**0.003**	1.156	1.052–1.270
HbA1c	0.765	0.103	**<0.001**	2.149	1.758–2.627
Sex	−0.097	0.234	0.679	0.908	0.574–1.435
Adropin	−1.981	0.220	**<0.001**	0.995	0.992–0.996
ET-1	0.986	0.149	**<0.001**	1.084	1.042–1.128
oxLDL	1.652	0.242	**<0.001**	1.024	1.017–1.031
MMP-2	0.980	0.139	**<0.001**	1.021	1.013–1.028
VEGFA	−0.249	0.118	0.692	0.999	0.994–1.004

Binary logistic regression has been performed. OR, odds ratio; *p* value < 0.05 is considered significant.

**Table 5:** Multivariable logistic regression models evaluating the association of circulating biomarkers with early vascular aging.

Biomarker	Model 1 (Crude) OR (95% CI)	*p* Value	Model 2 OR (95% CI)	*p* Value	Model 3 OR (95% CI)	*p* Value	Biomarker	Model 1 (Crude) OR (95% CI)	*p* Value	Model 2 OR (95% CI)	*p* Value	Model 3 OR (95% CI)	*p* Value
Adropin (per 100 pg/mL)	0.606 (0.548–0.740)	**<0.001**	0.495 (0.366–0.670)	**<0.001**	0.405 (0.331–0.670)	**<0.001**	Adropin	0.995 (0.994–0.997)	**<0.001**	0.993 (0.990–0.996)	**<0.001**	0.991 (0.989–0.996)	**<0.001**
ET-1 (per 1 pg/mL)	1.014 (1.005–1.024)	**0.003**	1.015 (1.004–1.024)	**0.006**	1.017 (1.005–1.029)	**0. 005**	ET-1	1.014 (1.005–1.024)	**0.003**	1.017 (1.005–1.029)	**0.006**	1.017 (1.005–1.029)	**0.005**
oxLDL (per 10 ng/mL)	1.083 (0.768–1.524)	0.658	1.480 (0.923–2.389)	0.101	1.438 (0.886–2.303)	0.139	oxLDL	1.008 (0.974–1.043)	0.658	1.040 (0.992–1.091)	0.101	1.037 (0.988–1.087)	0.139
MMP-2 (per 10 ng/mL)	1.010 (0.932–1.094)	0.806	1.041 (0.942–1.138)	0.452	1.030 (0.942–1.115)	0.478	MMP-2	1.001 (0.993–1.009)	0.806	1.004 (0.994, 1.013)	0.452	1.003 (0.994–1.011)	0.478
VEGFA (per 10 ng/mL)	0.970 (0.914–1.030)	0.346	0.951 (0.877–1.030)	0.184	0.951 (0.886–1.010)	0.178	VEGFA	0.997 (0.991–1.003)	0.346	0.995 (0.987, 1.003)	0.184	0.995 (0.988, 1.001)	0.178

Binary logistic regression has been performed. OR, odds ratio; *p* value < 0.05 is considered significant. Model 1 (crude): biomarkers only; Model 2: Model 1 + age + sex + HbA1c; Model 3 (fully adjusted): Model 2 + smoking status + alcohol status + medication use.

## Data Availability

The data presented in this study are available on request from the corresponding author.

## Supplementary Materials

The following supporting information can be downloaded at: https://www.mdpi.com/article/10.3390/diseases14040140/s1, Supplementary Table S1: Comparison of biochemical, vascular aging indices and vascular aging markers between controls and T2DM participants; Supplementary Table S2: Effect sizes for three-group comparisons using the Kruskal–Wallis test; Supplementary Table S3: Sensitivity analysis of EVA prevalence using alternative control-residual thresholds; Supplementary Table S4: Prevalence of early vascular aging across controls and T2DM subgroups.; Supplementary Table S5: Sex-wise distribution of early vascular aging (EVA) versus normal vascular aging status in the overall cohort; Supplementary Table S6: Spearman correlations of adropin with vascular, glycemic, lipid, and biomarker variables in the overall cohort and EVA subgroup; Supplementary Table S7: Multicollinearity diagnostics for predictors included in the adjusted logistic regression model; Supplementary Table S8: Box–Tidwell assessment of linearity in the logit for continuous predictors; Supplementary Table S9: ROC analysis for normal vascular aging from early vascular aging.; Supplementary Table S10: ROC analysis for T2DM subgroups (Good control vs Poor control); Supplementary Figure S1: Flowchart of participant screening of control and type 2 diabetes mellitus groups for analysis; Supplementary Figure S2: Correlation heatmap of all parameters in the study cohort; Supplementary Figure S3: ROC curve for T2DM subgroups (Good control vs Poor control).

## Abbreviations

AHD	Antihypertensive drugs
AFib	Atrial fibrillation
BMI	Body mass index
BP	Blood pressure
cfPWV	Carotid-femoral pulse wave velocity
CI	Confidence interval
CVD	Cardiovascular disease
DBP	Diastolic blood pressure
DCA	Decision curve analysis
ENHO	Energy homeostasis-associated gene
ePWV	Estimated pulse wave velocity
ET-1	Endothelin-1
EVA	Early vascular aging
FNR	False negative rate
FPG	Fasting plasma glucose
FPR	False positive rate
HbA1c	Glycated hemoglobin
LR+	Positive likelihood ratio
MBP	Mean blood pressure
NO	Nitric oxide
NPV	Negative predictive value
OHA	Oral hypoglycemic agents
OPD	Outpatient department
PPV	Positive predictive value
PWV	Pulse wave velocity
SBP	Systolic blood pressure
T2DM	Type 2 diabetes mellitus
TNR	True negative rate
TPR	True positive rate
VA	Vascular aging
