# Supplementary material for: Adropin and Endothelin-1 as Complementary Signals Associated with Early Vascular Aging in Middle-Aged Type 2 Diabetes
Source: Diseases. 2026 Apr 9;14(4):140. doi: 10.3390/diseases14040140 (PMC13115351; doi:10.3390/diseases14040140)
Supplement: Supplementary file 1 [file diseases-14-00140-s001.zip › diseases-4200671-supplementary.pdf]

Supplementary Table S1: Comparison of biochemical, vascular aging indices and vascular aging markers between controls and T2DM participants

Parameter	Control (n=150)	T2DM (n=150)	*p*- value
FPG, mg/dL	91 (86,95)	132.5 (110,176)	**< 0.001**
PPPG, mg/dL	116 (110,123.25)	203 (151,254)	**< 0.001**
HbA1c, %	5.2 (5,5.4)	7.4 (6.6,9.3)	**< 0.001**
eAG	103 (97,108)	166 (142.5,220)	**< 0.001**
Total cholesterol, mg/dL	154 (130,181.5)	180 (161.75,216)	**< 0.001**
Triglycerides, mg/dL	97 (71,123.75)	120.5 (95,163.5)	**< 0.001**
HDL-C, mg/dL	46 (41,52)	43.5 (38,49)	**0.021**
LDL-C, mg/dL	112.5 (91,128.5)	124 (107,148.5)	**< 0.001**
VLDL-C, mg/dL	19 (14,25)	24 (19,33)	**< 0.001**
Non-HDL-C, mg/dL	115 (93.25,133)	134 (115,163)	**< 0.001**
TC/HDL-C	3.56 ± 0.82	4.19 ± 0.98	**< 0.001**
LDL-C/HDL-C	2.49 ± 0.73	2.93 ± 0.80	**< 0.001**
TGL/HDL-C	2.4 ± 1.03	3.29 ± 1.7	**< 0.001**
ePWV, m/s	7.21 (6.86,7.7)	8.02 (7.39,8.49)	**< 0.001**
QRISK3, %	7.05 (4.1,8.5)	16.71 (14.2, 19.3)	**< 0.001**
Adropin, pg/mL	834.24 (653.4, 1884.9)	245.27 (119.85,528.74)	**< 0.001**
ET-1, pg/mL	1.29 (1.16,1.43)	5.59 (3.03,7.78)	**< 0.001**
oxLDL, ng/mL	41.87 (29.61,53.1)	102.5 (84,130.16)	**< 0.001**
MMP-2, ng/mL	9.79 (7.59,13.7)	57.08 (25.5,83.21)	**< 0.001**
VEGFA, ng/mL	48.79 (29.64,63.29)	39.48 (14.3,89.61)	0.587

p value < 0.05 is considered significant; FPG-Fasting Plasma Glucose, PPPG-Postprandial Plasma Glucose, eAG-Estimated Average Glucose, HDL-C-High-Density Lipoprotein Cholesterol, LDL-C-Low-Density Lipoprotein Cholesterol, VLDL-C-Very-Low-Density Lipoprotein Cholesterol, ePWV-Estimated Pulse Wave Velocity, ET-1-Endothelin-1, oxLDL-Oxidized Low-Density Lipoprotein, MMP-2-Matrix Metalloproteinase-2, VEGFA-Vascular Endothelial Growth Factor A

Supplementary Table S2: Effect sizes for three-group comparisons using the Kruskal–Wallis test

Parameter	Kruskal–Wallis H	p value	ε²	Interpretation
FBS	213.100	<0.001	0.711	Large
PPBS	209.315	<0.001	0.698	Large
HbA1c	252.635	<0.001	0.844	Large
eAG	252.634	<0.001	0.844	Large
ePWV	84.520	<0.001	0.278	Large
TCHO	49.995	<0.001	0.162	Medium
TGL	37.012	<0.001	0.118	Medium
HDL	5.588	0.061	0.012	Small

LDL	19.398	<0.001	0.059	Small
VLDL	34.609	<0.001	0.110	Medium
non-HDL	36.548	<0.001	0.116	Medium
TC/HDL	41.142	<0.001	0.132	Medium
LDL/HDL	25.266	<0.001	0.078	Small
TG/HDL	36.376	<0.001	0.116	Medium
Adropin	124.324	<0.001	0.412	Large
ET-1	205.022	<0.001	0.684	Large
oxLDL	204.710	<0.001	0.683	Large
MMP2	175.479	<0.001	0.584	Large
VEGF	28.089	<0.001	0.088	Medium

Epsilon squared (ε^2) was calculated for Kruskal–Wallis tests as $\varepsilon^2 = (H - k + 1) / (n - k)$; with $k = 3$ and $n = 300$, this becomes $\varepsilon^2 = (H - 2) / 297$. Interpretation: small = 0.01, medium = 0.08, large = 0.26.

Supplementary Table S3: Sensitivity analysis of EVA prevalence using alternative control-residual thresholds

Threshold	Residual cutoff (m/s)	Controls n (%)	T2DM good control n (%)	T2DM poor control n (%)	Overall EVA n (%)
**R75**	0.193	40/150 (26.7)	40/66 (60.6)	81/84 (96.4)	161/300 (53.7)
**R80**	0.235	11/150 (7.3)	37/66 (56.1)	68/84 (81.0)	116/300 (38.6)
**R85**	0.289	10/150 (6.7)	20/66 (30.3)	60/84 (71.4)	90/300 (30.0)

Supplementary Table S4: Prevalence of early vascular aging across controls and T2DM subgroups.

Variable	Control (n=150)	T2DM–HbA1c <7 (n=66)	T2DM–HbA1c ≥ 7 (n=84)	Overall (N=300)	*p*-value
EVA (age-specific), n (%)	11 (7.33)	37 (56.1)	68 (80.95)	116 (38.6)	<0.001
Normal, n (%)	139 (92.66)	29 (43.9)	16 (19.04)	184 (61.4)	

Group differences for EVA prevalence tested by χ^2; p value < 0.05 is considered significant.

Supplementary Table S5: Sex-wise distribution of early vascular aging (EVA) versus normal vascular aging status in the overall cohort

Sex	Normal, n (%)	EVA, n (%)	Total (n)	χ^2 (df=1)	*p*-value
Male	93 (61.2)	59 (38.8)	152	0.025	0.876
Female	91 (61.5)	57 (38.5)	148		
Overall	184	116	300		

Sex differences for EVA prevalence were tested by χ^2; p-value < 0.05 is considered significant.

Supplementary Table S6: Spearman correlations of adropin with vascular, glycemic, lipid, and biomarker variables in the overall cohort and EVA subgroup

Panel	Variable	Overall population ρ	p value	EVA subgroup ρ	p value
A	ET-1	-0.546	<0.001	-0.439	<0.001
B	oxLDL	-0.421	<0.001	-0.216	0.003
C	MMP-2	-0.515	<0.001	-0.404	<0.001
D	VEGFA	0.157	0.006	-0.026	0.731
E	ePWV	-0.507	<0.001	-0.220	0.013
F	HbA1c	-0.603	<0.001	-0.435	<0.001
G	Total cholesterol	-0.129	0.025	-0.117	0.191
H	HDL-C	0.175	0.002	0.144	0.106
I	LDL-C	-0.089	0.124	-0.173	0.048
J	VLDL-C	-0.202	<0.001	-0.093	0.304
K	Triglycerides	-0.207	<0.001	-0.083	0.356
L	Non-HDL-C	-0.164	0.005	-0.177	0.048
M	QRISK3	-0.511	<0.001	-0.381	<0.001

Supplementary Table S7: Multicollinearity diagnostics for predictors included in the adjusted logistic regression model

Predictor	Tolerance	VIF
**ET-1**	0.833	1.200
**Adropin**	0.775	1.290
**oxLDL**	0.707	1.414
**MMP2**	0.720	1.389
**VEGFA**	0.928	1.078

Supplementary Table S8: Box–Tidwell assessment of linearity in the logit for continuous predictors

Predictor interaction term	B	p value
Adropin ln	-0.161	0.448
ET1 ln	0.046	0.330
oxLDL ln	-0.556	0.625
MMP2 ln	0.190	0.810
VEGFA ln	-0.397	0.506

Supplementary Table S9: ROC analysis for normal vascular aging from early vascular aging.

Parameter	Cut-off	Youden's J	Sensitivity % (TPR)	Specificity % (TNR)	FPR %	FNR%	PPV%	NPV%	LR+	Accuracy %
Adropin (pg/mL)	677	0.631	76.4	86.7	13.3	23.6	78.4	82.7	5.74	81.6
ET-1 (pg/mL)	3.13	0.471	68.5	78.6	21.4	31.5	66.9	79.8	3.20	74.7
Adropin + ET-1 (pred)	0.607	0.660	78.7	87.	12.7	21.3	79.6	86	6.2	84

Binary logistic regression was used to derive a combined predictor of Adropin and Endothelin-1; FPR- False positive rate, FNR- False negative rate, PPV- Positive predictive value, NPV- Negative predictive value, LR+- Positive likelihood ratio. p-value < 0.05 is considered significant.

Supplementary Table S10: ROC analysis for T2DM subgroups (Good control vs Poor control)

Parameter	Cut-off	Youden's J	Sensitivity % (TPR)	Specificity % (TNR)	FPR %	FNR%	PPV%	NPV%	LR+	Accuracy %
Adropin (pg/mL)	502	0.574	95.2	62.1	37.9	4.8	76.4	90.9	2.51	80.7
ET-1 (pg/mL)	3.44	0.761	98.8	77.3	22.7	1.2	84.9	98	4.35	89.4

FPR- False positive rate, FNR- False negative rate, PPV- Positive predictive value, NPV- Negative predictive value, LR+- Positive likelihood ratio. p-value < 0.05 is considered significant.

Supplementary Figure S1: Flowchart of participant screening of control and type 2 diabetes mellitus groups for analysis.

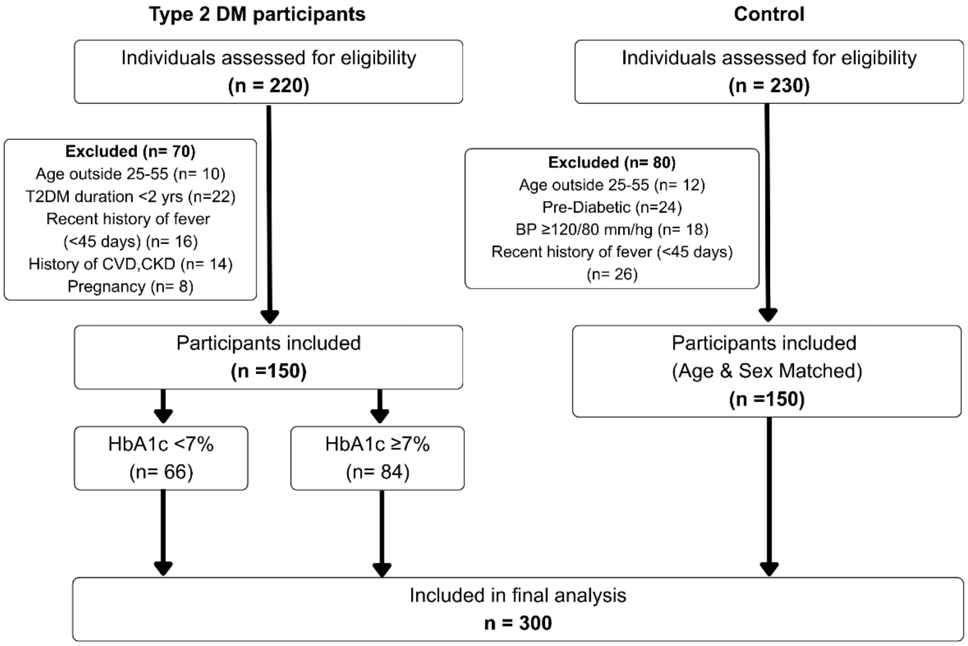

Supplementary Figure S2: Correlation heatmap of all parameters in the study cohort

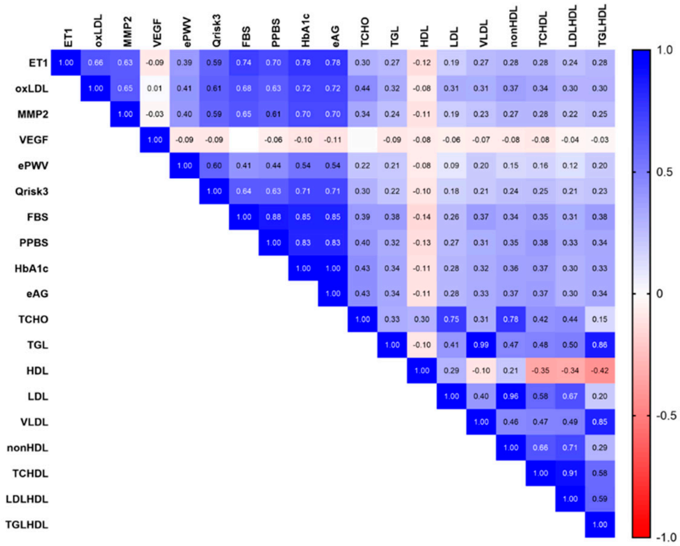

Pair	Raw p value	FDR result
ePWV – LDL/HDL	0.041	Significant
PPBS – HDL	0.029	Significant
ET-1 – HDL	0.034	Significant
FBS – HDL	0.013	Significant
TCHO – TGL/HDL	0.012	Significant
ePWV – non-HDL	0.009	Significant
ePWV – TC/HDL	0.005	Significant
Qrisk3 – LDL	0.002	Significant

Spearman correlation coefficients are shown. P values were adjusted for multiple testing using the Benjamini–Hochberg false discovery rate procedure across all unique pairwise correlations in the matrix. Associations remaining significant after FDR correction

Supplementary Figure S3: ROC curve for T2DM subgroups (Good control vs Poor control)

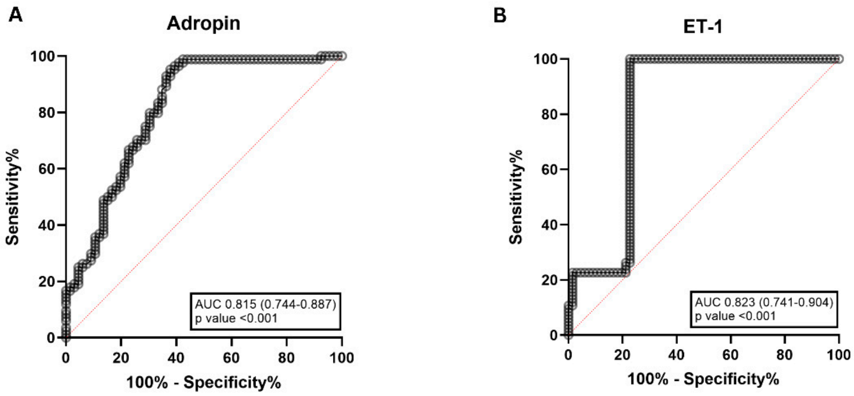

ROC curve of (A) Adropin, (B) ET-1.
